# The Best of Both Worlds: The Benefits of Open-specialized and Closed-diverse Syndication Networks for New Ventures’ Success

**DOI:** 10.1177/0001839216637849

**Published:** 2016-03-02

**Authors:** Anne L.J. Ter Wal, Oliver Alexy, Jörn Block, Philipp G. Sandner

**Affiliations:** 1 Imperial College Business School, London; 2 TUM School of Management, Technische Universität München; 3 Universität Trier and Erasmus Research Institute of Management (ERIM), Erasmus University Rotterdam (EUR); 4 Frankfurt School of Finance & Management and TUM School of Management, Technische Universität München

**Keywords:** social capital, network, startups, brokerage, structural holes, closure, network diversity, actors’ knowledge similarity, information redundancy, venture capital

## Abstract

Open networks give actors non-redundant information that is diverse, while closed networks offer redundant information that is easier to interpret. Integrating arguments about network structure and the similarity of actors’ knowledge, we propose two types of network configurations that combine diversity and ease of interpretation. Closed-diverse networks offer diversity in actors’ knowledge domains and shared third-party ties to help in interpreting that knowledge. In open-specialized networks, structural holes offer diversity, while shared interpretive schema and overlap between received information and actors’ prior knowledge help in interpreting new information without the help of third parties. In contrast, actors in open-diverse networks suffer from information overload due to the lack of shared schema or overlapping prior knowledge for the interpretation of diverse information, and actors in closed-specialized networks suffer from overembeddedness because they cannot access diverse information. Using CrunchBase data on early-stage venture capital investments in the U.S. information technology sector, we test the effect of investors’ social capital on the success of their portfolio ventures. We find that ventures have the highest chances of success if their syndicating investors have either open-specialized or closed-diverse networks. These effects are manifested beyond the direct effects of ventures’ or investors’ quality and are robust to controlling for the possibility that certain investors could have chosen more promising ventures at the time of first funding.

Social capital and network theorizing shape our thinking about how individuals and organizations derive information advantages from the networks in which they are embedded (Kwon and Adler, 2014). There is a strong research tradition in management around the structural dimension of social capital (Kilduff and Brass, 2010), with a central debate revolving around which network structures provide greater information advantages: open, sparse structures rich in structural holes or closed, dense structures with many shared third-party ties (e.g., Ahuja, 2000; Gargiulo and Benassi, 2000; Burt, 2005). Open networks are thought to yield information advantages in the form of access to diverse information (Burt, 2004), because information obtained from mutually unconnected parties—that is, from across a structural hole—is likely to be non-redundant. In contrast, being embedded in closed, densely connected networks is believed to allow access to detailed and in-depth information that is easier to interpret. In closed networks, pairs of actors have many joint third-party connections that induce trust in and commitment to their relationship and that create information redundancy, which in turn allows for greater capacity to transmit information (Uzzi, 1996; Reagans and McEvily, 2003; Aral and Van Alstyne, 2011). The question is how network actors can access diverse information that they can also effectively interpret.

Generally, actors in open networks have access to diverse information but also may have limited means to interpret it (Shipilov and Li, 2008). Interpretation may be particularly challenging because information providers in open networks may give little contextual information that could help interpretation (Aral and Van Alstyne, 2011) and have little incentive to be accurate and unbiased (Schilling and Fang, 2014). The literature on open networks and structural holes has been largely silent about how actors in open networks effectively absorb the diverse information they access. It treats the interpretive ability of actors as exogenously determined rather than as a function of the network’s structure and composition.

Conversely, actors in closed networks can more easily interpret information yet may lack requisite diversity. Uzzi (1996) argued that the repeated interactions within closely tied groups of actors typical of closed networks carry the risk of a strong convergence of ideas and insights that, combined with a lack of inflow of information from sparse connections, can lead to a lack of information diversity. Although subsequent research has advanced our understanding of how diversity in closed networks can help overcome such problems (Reagans and McEvily, 2003; Rodan and Galunic, 2004; Fleming, Mingo, and Chen, 2007), our understanding of the mechanisms that allow shared third parties to contribute to the interpretation of diverse knowledge may be incomplete. Research has emphasized the indirect benefits provided by shared third parties, such as induced commitment and trust (Reagans and McEvily, 2003; Tortoriello and Krackhardt, 2010; Tortoriello, Reagans, and McEvily, 2012), but has neglected that third parties can contribute more directly through the collective interpretation of the information they also receive from the same shared alter.

To integrate into network theory the ability of network actors to interpret information, we must consider both network structure and actors’ knowledge similarity to fully understand how actors obtain and effectively interpret diverse information. Building on research on network range (Reagans and McEvily, 2003), knowledge heterogeneity (Rodan and Galunic, 2004; Fleming, Mingo, and Chen, 2007), and boundary spanning (Hargadon and Sutton, 1997; Tortoriello and Krackhardt, 2010), we define actors’ knowledge similarity as the extent to which network actors specialize in the same knowledge domains.

We argue that actors can most effectively access and interpret diverse information from two prototypical network configurations. First, closed-diverse networks feature numerous shared third-party ties among dissimilar actors. In this case, access to diverse information is enabled by the heterogeneity of the actors’ knowledge domains, and shared third-party connections are a conduit that allows corroboration of potentially different interpretations of that diverse information via triangulation (Brown and Duguid, 1998; Gavetti and Warglien, 2015). Closed-diverse networks may offer advantages over both closed-specialized networks, which lack information diversity, and open-diverse networks, which provide information that cannot be interpreted effectively. Second, open-specialized networks are sparse structures among actors with similar specializations. In those networks, the focus on similar knowledge domains is accompanied by shared interpretive schema (Simon and Feigenbaum, 1964; Simon, 1966; Brewer and Nakamura, 1984) and redundancy between the information received and the receiver’s prior information (Shannon and Weaver, 1948), helping actors interpret new information without the help of third parties. Open-specialized networks are likely to offer benefits over open-diverse networks, which lack shared interpretive schema, and closed-specialized networks, in which diversity is compromised. Thus open-specialized and closed-diverse networks should provide the best of both worlds by combining the diversity stemming from the network structural dimension with the ability to interpret information deriving from the actors’ knowledge similarity dimension, and vice versa.

We test our arguments by studying the value of investors’ social capital to the early-stage ventures in which they invest. Ventures that receive investor funding gain not only from access to those investors’ financial and human capital but also because the investors act as important channels of information that can give a new venture a competitive edge (Hochberg, Ljungqvist, and Lu, 2007; Hallen, 2008; Lungeanu and Zajac, 2015; Pahnke, Katila, and Eisenhardt, 2015). Investors and the networks they build through syndication constitute an important form of social capital that new ventures can exploit to sustain profitability and long-term survival (Stuart, Hoang, and Hybels, 1999; Sorenson and Stuart, 2001). New ventures benefit from their investors’ access to diverse insights from across various domains, provided that the investors are able to interpret this information and apply it to the specific domain of the venture. We examine how the information advantages that newly funded early-stage ventures obtain from the syndication networks of their first-round investors contribute to their success in attracting additional funding.

## The Value of Investors’ Social Capital to Funded Ventures

Syndication relationships are a key ingredient of investors’ social capital, as prior syndication relationships allow investors to build networks that offer informational advantages to support their investment decisions (Bygrave, 1987; Hsu, 2006; Dimov and Milanov, 2010; Milanov and Shepherd, 2013; Liu and Maula, 2015). Embeddedness in syndication networks offers investors information about new investment opportunities that is shared within a high-trust environment and is not accessible to those outside the network (Sorenson and Stuart, 2001). For example, venture capitalists with high social capital have a higher willingness to invest large sums in startups because their privileged access to information on a venture’s quality lowers the perceived risk of the investment and increases the evaluation of future cash flows (Alexy et al., 2012).

The social capital that investors build through past syndication experience is an important asset for both the investors and the invested venture (Hochberg, Ljungqvist, and Lu, 2007; Hallen, 2008). After a new venture’s first investment round, its investors typically assume an advisory role, which makes their accumulated social capital from past investment activities available to the venture. This fosters the venture’s development and increases the returns to the investors (Stuart, Hoang, and Hybels, 1999; Sorenson and Stuart, 2001). For startups, investors’ network resources are a form of second-order social capital that provides key advantages (Galunic, Ertug, and Gargiulo, 2012). Investors typically need to be actively involved to help early-stage ventures grow, but investors’ own resources and knowledge may be insufficient for them to provide high-quality advice. Several studies have shown that the number of an investor’s network connections positively affects the performance of the funded venture (e.g., Hochberg, Ljungqvist, and Lu, 2007), ultimately increasing the likelihood of a successful exit (Shane and Stuart, 2002; Hsu, 2006; Fitza, Matusik, and Mosakowski, 2009). Two aspects of investors’ social capital may be particularly valuable to their portfolio companies: (1) the informational diversity in their network and (2) their ability to interpret how that information applies to the specific context of the venture.

First, the value of investors’ social capital to their portfolio firms is a function of their access to diverse information on which they can base their advice (Lungeanu and Zajac, 2015). Individual investors might have deep sector-specific and location-specific expertise, but diversity of expertise from across one’s own domain is also important in this context (Bellavitis, Filatotchev, and Kamuriwo, 2014). Syndication with other investors exposes investors to unfamiliar information and insights into other sectors and locations (Sorenson and Stuart, 2001; Hochberg, Ljungqvist, and Lu, 2007; Liu and Maula, 2015). Although investors tend to syndicate with others with similar industry profiles (Sorenson and Stuart, 2001), heterogeneous syndication ties are formed every time investors with different backgrounds and portfolios are attracted to the same target companies (Sorenson and Stuart, 2008), when they bring complementary resource endowments to the investment (Hochberg, Lindsey, and Westerfield, 2015), or when investors decide to alter their investment policies on the basis of inconsistent performance feedback from prior investments (Baum et al., 2005). Insights from one setting or knowledge domain can potentially be valuable in some other setting in providing a new solution unknown in that setting (Hargadon and Sutton, 1997; Hsu and Lim, 2014) or bringing a new perspective to a problem (Rosenkopf and Nerkar, 2001; Perry-Smith, 2006).

Second, the syndicate’s ability to interpret diverse information meaningfully cannot be taken for granted. There may be interpretive barriers to understanding the information and assessing its value (Bruner, Goodnow, and Austin, 1956; Simon and Feigenbaum, 1964; Dougherty, 1992), which may limit investors’ ability to integrate information from various sources to generate new insights (Simon, 1966; Mors, 2010; Wadhwa, Phelps, and Kotha, 2016). The quality of the advice provided to portfolio ventures will depend on investors’ ability to interpret the information obtained and to assess how it could be applied to the venture’s specific setting, which may in turn depend on whether the investors’ social capital comes from open versus closed networks, and specialized versus diverse networks.

### Network Closure: Open vs. Closed Syndication Networks

In the network literature, network structure is argued to shape the informational advantages actors can derive from their social capital (e.g., Zukin and DiMaggio, 1990; Granovetter, 1992), triggering debate over which network structures provide the greatest informational advantages. The debate revolves around a defining aspect of network structure: the level of redundancy in the information that actors can access from the network (Burt, 1992). Redundancy is a function of the degree of closure among a focal actor’s direct ties. An actor connected to two alters who are directly connected to each other will likely access redundant information (Coleman, 1990; Uzzi, 1996). If the alters are unconnected—if the focal actor spans a structural hole—the information they provide to the focal actor is likely to be non-redundant (Burt, 1992, 2004). Both redundant and non-redundant information provide important advantages, and both are considered pivotal to social capital. Some researchers suggest that these advantages are not necessarily irreconcilable. For example, the timing might differ in that advantages from structural holes may emerge more quickly and be more short-lived than those from closure (Soda, Usai, and Zaheer, 2004; Zaheer and Soda, 2009; Baum, McEvily, and Rowley, 2012). Also, the advantages of open and closed networks may operate at different levels in the network, for example within or beyond teams (Zaheer and Soda, 2009). Finally, open and closed structures might coexist concurrently and at the same level in a network (Oh, Chung, and Labianca, 2004; Schilling and Phelps, 2007; Reagans and McEvily, 2008). Although these studies suggest important contingencies related to the value of structural holes and closure, they do not address how networks can combine the advantages of redundant information in closed networks and the advantages of non-redundant information in open networks.

On the one hand, information redundancy associated with closed networks is considered advantageous because it eases interpretation. There is a high likelihood that the same information may reach network actors via multiple routes in the network. Given that various providers may communicate the information differently, redundancy enables information receivers to cross-check or triangulate the information (Krackhardt, 1999; Tortoriello and Krackhardt, 2010). As Shannon and Weaver (1948) argued, redundant information reduces the probability of interpretation error because information receivers may understand different aspects of a particular message from different sources. In certain circumstances, new information may be judged credible only if confirmed by multiple sources (Centola and Macy, 2007). The interpretation of information in closed networks is eased also by the increased channel bandwidth of ties in such networks (Aral and Van Alstyne, 2011): information richness and detail are enhanced because two parties are more committed to the exchange if they have a common third party (Reagans and McEvily, 2003). The increased transmission capacity allows for the exchange of sensitive and complex information (Fleming, Mingo, and Chen, 2007).

On the other hand, the flows of non-redundant information typical of open networks may be advantageous because they tend to incorporate greater information diversity, which can help change or challenge existing perspectives (Burt, 1992, 2004). Network actors who connect otherwise disconnected individuals gain access to information that is diverse and that they can exploit to their own advantage (Burt, 1992) or use instrumentally to establish collaboration between previously disconnected parties (Obstfeld, 2005; Lingo and O’Mahony, 2010).

A theoretical tension related to the value of open versus closed networks arises because the ease-of-interpretation advantage of redundant information in closed networks is lacking in open networks, and the diversity advantage of non-redundant information in open networks is lacking in closed networks. The literature on open networks—with the exception of process studies on brokerage (Obstfeld, 2005; Lingo and O’Mahony, 2010)—does not elaborate how actors interpret information (Burt, 2010). In the structuralist tradition of research on open networks, actors accessing diverse information from structural holes are implicitly ascribed the ability to process it effectively and use it to their advantage. But actors receiving diverse information in the absence of redundancy from overlapping ties and without the ability to triangulate the information to ease its interpretation may not be able to process and use it effectively (Coleman, 1990; Shipilov and Li, 2008). The assumption of actors’ interpretive ability is particularly problematic because information providers in open networks have few incentives to expend effort and time on information exchange, which leads to reduced richness and detail in the information provided (Aral and Van Alstyne, 2011), and they lack pressure from shared third parties not to behave opportunistically (Burt, 2005; Shipilov and Li, 2008; Tortoriello and Krackhardt, 2010). In contrast, closed networks can suffer from a lack of non-redundancy. From a purely structuralist perspective, actors in closed networks have access to rich, detailed information they can effectively interpret but that potentially lacks diversity (Uzzi, 1997; Gargiulo and Benassi, 2000). Repeated interactions among close-knit groups of actors could lead to the convergence of ideas and insights that, combined with a lack of inflow from sparse connections, reduces information diversity and introduces the risk of groupthink. Network actors in closed networks can fail to challenge collectively held beliefs and become trapped in their own nets (Uzzi, 1997; Gargiulo and Benassi, 2000).

In the context of social capital in syndication networks, open networks occur if only some pairs of investors have prior syndicated investments. These networks contain non-redundant, diverse perspectives on the elements contributing to venture success, but it may be difficult for the investors to make sense of how insights from non-shared investments might apply in a new context. Closed networks among groups of investors occur if most actor pairs have co-invested in the past. In these networks, there is redundant information on what was or was not successful in past portfolio companies, which facilitates the formation of shared beliefs among network actors about why ventures succeed or fail, but it introduces the risk of taken-for-granted views and ingrained assumptions going unchallenged.

### Actors’ Knowledge Similarity: Specialized vs. Diverse Syndication Networks

A network structural perspective alone does not explain how network actors gain access to diverse and interpretable information. The level of information diversity and the ability of network actors to interpret that information depend also on the heterogeneity of these actors’ knowledge. The interplay between network structure—open versus closed networks—and actors’ knowledge similarity—diverse versus specialized networks—offers a solution to the puzzle of how actors can access information that is both diverse and interpretable.

A number of studies suggest that the relative similarity of the actors in a network—in addition to its structure—facilitates access to diverse information and affects actors’ ability to interpret it. We build on the concepts of network range (Reagans and McEvily, 2003; Tortoriello, Reagans, and McEvily, 2012) and knowledge heterogeneity (Rodan and Galunic, 2004) to emphasize that the information value of social capital depends not only on network structure but also on the knowledge properties of the network actors. We define actors’ knowledge similarity as the extent to which network actors are specialized relative to one another. In specialized networks, actors focus on similar knowledge domains, and most of the information circulating tends to fall within those domains. Diverse networks have actors specialized in dissimilar knowledge domains and thus can provide access to unfamiliar information. In the context of syndication networks, specialization occurs when the investors in a network are similar in terms of the sectoral focus of their past investments. Actors’ knowledge similarity differs from network range in that the former captures not the dispersion of the knowledge in the network but the extent to which the network actors are similar (Harrison and Klein, 2007). If each actor spans many domains and these domains are the same for all actors, the range (dispersion) is high and actors’ similarity is also high.

**Figure 1.**
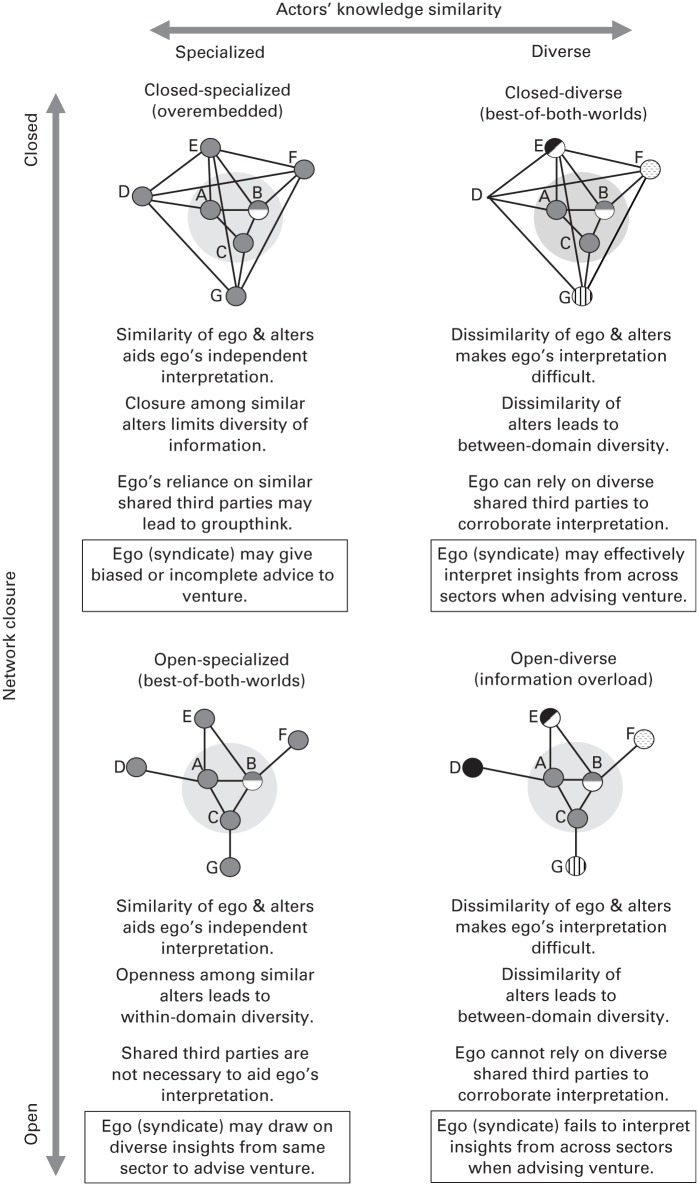
The interplay between network structure and actors’ knowledge similarity in explaining informational advantage.* * Node shadings/patterns are indicative of the sectoral focus of actors’ past investments. Nodes with multiple shadings/patterns have prior investments in multiple sectors.

Building on the argument that ventures thrive when their investors advise them with diverse information they can sensibly interpret and apply to the venture’s context (Lungeanu and Zajac, 2015; Wadhwa, Phelps, and Kotha, 2016), the level of actors’ knowledge similarity should moderate the relationship between syndication network closure and venture success. Specifically, actors’ knowledge similarity should also influence the level of information redundancy or non-redundancy in the network. Thus including knowledge similarity in network theorizing is key to determining how networks can provide access to information that is both diverse and interpretable. Figure 1 depicts the network configurations that combine the network structure and actors’ knowledge similarity properties of a syndication network and summarizes the value that these configurations of investors’ social capital embody for the ventures in which they invest.

### The Value of Closed-diverse Syndication Networks

The first type of syndication network (figure 1, upper right) that provides diverse, interpretable information has a high level of network closure among diverse network actors. This corresponds to a situation in which the focal investors and their past syndication partners have, in different compositions, regularly co-invested in the past but are dissimilar in terms of their aggregate profile of subsectors of past investments. That is, in addition to having co-investments that produce high levels of closure, each of the network actors has been involved in additional investments in other sectors. The flows of information in these networks are likely to be rich because the presence of shared third parties motivates the actors to spend time and effort on the exchange (Reagans and McEvily, 2003).

Such closed-diverse networks offer a diversity advantage because each actor can bring to the table insights into best practices, ongoing trends, and developments from various sectors. This information is valuable in providing a perspective on how domain-specific knowledge relates to knowledge in other domains (Reagans and Zuckerman, 2001; Rosenkopf and Nerkar, 2001; Fleming and Waguespack, 2007). Exposure to insights from unfamiliar domains can stimulate investors to reflect on their own knowledge domain, challenge taken-for-granted views, and broaden the range of alternatives beyond those common to the domain (e.g., Hargadon and Sutton, 1997; Perry-Smith, 2006; Grégoire, Barr, and Shepherd, 2010).

Closed-diverse networks also offer built-in advantages for interpreting diverse information. Information from outside the venture’s domain can be difficult to interpret (Dougherty, 1992; Bechky, 2003; Tortoriello and Krackhardt, 2010), and applying such information to a specific context is a non-trivial task (Mors, 2010). Actors’ embeddedness in a closed-diverse network can ease the interpretation of diverse information in two ways. First, two parties exchanging information tend to be more committed to spending time and effort on the exchange if they have common third-party connections (Reagans and McEvily, 2003). Awareness of common connections increases trust in the relationship and discourages the willful provision of incorrect information (Coleman, 1990; Walker, Kogut, and Shan, 1997; Tortoriello and Krackhardt, 2010; Rosenbaum, Billinger, and Stieglitz, 2014). Investors may more easily interpret diverse information from closed networks because alters expend more effort on communicating insights from other settings in greater detail.

Second, the presence of shared third parties enables redundancy in interpretive cues through triangulation, which can make interpretation easier. In closed triadic structures, actors may receive the original information directly from the information provider, as well as others’ interpretations of it via shared third parties who likely received the same information from the provider. Paying attention to other people’s interpretations of the same information improves one’s own interpretation (Weick and Roberts, 1993; Gavetti and Warglien, 2015). By uncovering overlaps and differences in interpretation, focal actors can make inferences about the accuracy of their own interpretations. This triangulation process is effective specifically in diverse networks. Interpretation through interaction is a distributed cognition process (Michel, 2007) that may be particularly effective with heterogeneous alters who are likely to interpret the same information differently and thus to offer the focal actor different versions of the same story to triangulate. Thus groups of interconnected diverse actors provide a platform for the collective interpretation of diverse information (Brown and Duguid, 1998; Gavetti and Warglien, 2015; Tortoriello, McEvily, and Krackhardt, 2015).

In the context of investor syndication networks, investors may collectively interpret the implications of new trends in emerging sectors for ventures in their portfolio, perhaps exchanging and discussing the business plans of various firms in the process (Bygrave, 1987; Hsu, 2006; Milanov and Shepherd, 2013; Liu and Maula, 2015). The combined diversity and interpretation advantages of closed-diverse networks imply that syndicates will be able to formulate high-quality advice for the venture based on diverse information from various sectors that trusted third parties have helped interpret and apply to this venture’s particular subsector.

Syndicates with closed-diverse networks can provide more valuable information to the venture than syndicates with closed-specialized networks, which lack access to a variety of information from outside the venture’s domain (figure 1, upper left). Members of close-knit groups can suffer from groupthink, hesitating to explore new ideas, as they prefer the prevailing schema shared within the group (Janis, 1972; Wang et al., 2013). The advantage of the information richness typical of closed networks is undermined by the lack of non-redundant information. The term overembeddedness was coined by Uzzi (1997: 58) to describe such situations, in which “all firms in a network are connected through embedded ties, [which] can reduce the flow of new and novel information into the network because . . . there are few or no links to outside actors who can potentially contribute innovative ideas.” We extend the concept of overembeddedness to include the lack of inflow of novel ideas due to excessive levels of actors’ knowledge similarity in the network. The downsides associated with scarce connections to parties outside the dense cliques in the network are exacerbated if the respective actors are very similar. Triangulation does not work in closed-specialized networks and can even lead the actors to believe that incorrect information is correct. Cross-checking information via third parties who both received the original information from the same source and interpreted it from the same perspective is unlikely to lead to interpretation differences that can be triangulated. Hence inaccurate or outdated information may remain unchallenged. Investors in a closed network of actors with similar investment profiles are likely to give their ventures incomplete and possibly biased advice; they have access to only a limited view of best practices, trends, and developments in a specific sector, which may lead to advice based on shared myths that are not challenged by the inflow of diverse information. This limitation will be reflected by the venture’s lower levels of success.

Closed-diverse networks also offer advantages over open-diverse networks (figure 1, lower right), in which the value and accuracy of the information cannot be judged effectively because there are no opportunities for triangulation through shared parties so information overload may become a problem. There is also a risk that syndicates with open-diverse networks may advise ventures based on information whose application to a particular sector or venture they cannot adequately assess. Investors’ misinterpretations of how insights from one sector may or may not transfer to another sector could damage the venture’s probability of success. Thus we predict that:

**Hypothesis 1a (H1a):** Venture success is more likely if the investing syndicate has a closed-diverse network rather than a closed-specialized or open-diverse network.

### The Value of Open-specialized Syndication Networks

The second type of syndication network providing diverse, interpretable information is a network with a low level of network closure among similarly specialized network actors (figure 1, lower left). In open-specialized networks, actors focus on the same knowledge domains but form networks rich in structural holes. This corresponds to a situation in which focal investors and their past syndication partners have little past experience of co-investment despite their focus on the same subsectors. Actors in such networks have substantial incentives to share information despite the absence of shared third parties, although information depth and richness may be less. As with any other prior tie, the sparse connections in open networks result from a previous shared commitment, in our context a shared investment, which makes it more likely the two parties will form a bond of trust and be ready to share information with each other (Sorenson and Stuart, 2001). Information sharing in open-specialized networks is likely also because similarity breeds trust. Gulati and Sytch (2008: 182) showed that “organizations that are more similar to each other can derive greater stocks of trust from [their] joint history compared to more heterogeneous sets of partners.”

Analogous to the value of closed-diverse networks, the value to ventures of open-specialized syndication networks is based on the combination of non-redundancy, which brings information diversity, and redundancy, which facilitates interpretation. Open-specialized networks have a diversity advantage: the open structure safeguards the syndicate’s access to diverse, non-redundant information because each network actor brings insights and experiences from different investments, possibly in different geographic contexts (Lingo and O’Mahony, 2010). Alters likely have different views about the sector-specific ingredients for a venture’s success, helping investors to challenge and update insights obtained from their specific experiences and to prevent local bias (Jääskeläinen and Maula, 2014).

Open-specialized networks also have an ease-of-interpretation advantage. Above we argued that, when considering structural arguments only, it is not clear how actors in open networks can overcome the hurdles to interpretation associated with information that is diverse, non-redundant, and potentially unfamiliar (Shipilov and Li, 2008) and is not as rich as information from closed networks (Aral and Van Alstyne, 2011). We propose that actors in open networks can overcome these barriers if their networks are specialized. Information receivers will be better able to interpret information if it comes from similar others and relates to a familiar domain (Bruner, Goodnow, and Austin, 1956). The receiving actor’s prior knowledge creates redundancy with the received information, which makes it easier to interpret and makes it understandable even if it is incomplete or of poor quality (Shannon and Weaver, 1948). Information receivers in open-specialized networks will have both the interpretive schema to assess the meaning of information relative to what they already know and the evaluation abilities to judge its relation to prior knowledge (Simon and Feigenbaum, 1964; Simon, 1966; Brewer and Nakamura, 1984; Hwang, Singh, and Argote, 2014). Thus, relative to information from outside the investor’s domain, the need for triangulation to interpret within-domain information and the need for a sounding board to understand how it applies to the focal venture are much reduced. This allows the information-receiving investors to interpret information without the help of third parties (Hwang, Singh, and Argote, 2014) and to advise ventures on the basis of diverse, properly interpreted information.

Investors in open-specialized networks have access to a variety of insights into how firms can succeed in a specific subsector. The views of the various prior partners in their network will likely differ because of the structural holes among them, so these partners have not come to a consensus on what makes a successful business in the particular subsector. The familiarity of the information-receiving investors with the sector, and their prior knowledge in that domain, ensure that they can interpret and apply divergent views to decide the best course for the focal venture even when the information accessed lacks depth and detail and when there are no shared third parties on whose interpretation they can rely. The combined diversity and interpretation advantages of open-specialized networks imply that syndicates in such networks will be able to formulate high-quality advice for the venture based on diverse information from within the sector, which was effectively interpreted and applied by tapping into prior knowledge of the domain.

Syndicates with open-specialized networks offer greater information value to a venture than those with open-diverse networks (figure 1, lower right) in which there is little or no redundancy between the information received and the receiver’s prior knowledge and in which actors have no shared interpretive schema. Borrowing from research on information processing (see, e.g., Bruner, Goodnow, and Austin, 1956; Simon, 1974; Sweller, 1988) and recent research on networks (Mariotti and Delbridge, 2012), we use the term information overload to describe a situation in which actors in an open and highly diverse network lack the ability to correctly process the huge diversity of information arising from both open structures and actors’ knowledge diversity. In addition to the actors lacking the carrying capacity to deal with the volume of diverse information (Simon, 1956; Hansen and Haas, 2001; Hwang, Singh, and Argote, 2014), their ability to absorb and interpret it may be compromised (Simon and Feigenbaum, 1964; Simon, 1966; Ghosh and Rosenkopf, 2014). The absence of shared third parties to help corroborate diverse information, as well as the reduced ability to make independent judgments because of mismatches in investors’ interpretive schema and lack of prior knowledge, implies that investors in open-diverse networks are not able to interpret diverse information meaningfully. Investors with an open network of actors with dissimilar investment profiles may provide investors with unsound, speculative advice based on a broad range of insights from unfamiliar sectors, which may have been misinterpreted and applied erroneously to the context of the focal venture. Thus we argue that investors in open-diverse networks are unable to formulate coherent advice that will be of value to their ventures. Open-specialized syndication networks are also superior to closed-specialized networks, which lack the requisite variety to help investors update sector-specific insights and challenge assumptions. Thus we predict:

**Hypothesis 1b (H1b):** Venture success is more likely if the investing syndicate has an open-specialized network rather than an open-diverse or closed-specialized network.

### Information Redundancy and Network Configuration

**Figure 2.**
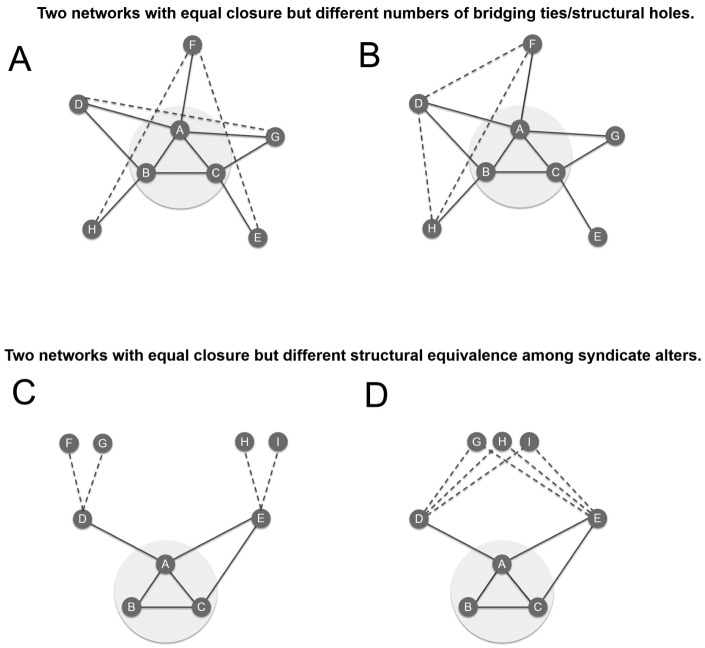
Structural holes and structural equivalence at equal levels of closure.

Both closed-diverse networks and open-specialized networks combine the ease-of-interpretation advantages of information redundancy and the diversity advantages of non-redundancy. First, this argument is based on the assumption that information redundancy is a function of network closure and actors’ knowledge heterogeneity. Regarding the former, we assume that open networks are rich in structural holes, which are the basis of non-redundancy: investors access diverse information through bridging ties across structural holes between parties with no mutual prior syndication relations. Although structural holes typically are pervasive in open networks, as shown in figure 2, diagrams A and B, the number of bridging ties across structural holes in relatively open networks also depends on the distribution of the alter–alter ties (dashed lines) in the network. In diagram A, syndicate ABC has no bridging ties, because there is no single alter with whom the syndicate has no third party in common. In diagram B, syndicate ABC has two bridging ties. For both alters E and G there are no other alters in common with the syndicate. Networks with fewer (or more) alter–alter ties always have lower (or higher) levels of redundancy, but bridging ties may more accurately capture the level or lack of redundancy in the network structure than the characterization as open or closed.

Second, information redundancy based on network structure depends not only on the interconnectedness of the alters but also on the extent to which these alters are structurally equivalent (Lorraine and White, 1971; Reagans and Zuckerman, 2008)—that is, the extent to which they are tied to the same third parties. Two unconnected investors may bring little diversity to the syndicate if they are informed by the same investors in the second shell of the network from the syndicate’s perspective (see figure 2, diagrams C and D). In diagram C, members D and E—both alters of the syndicate ABC—are not structurally equivalent because they have different third-party ties, F–G and H–I (shown as dashed lines), respectively. In diagram D, members D and E are fully structurally equivalent because they share the same third parties, G, H, and I. Closure captures the redundancy of information that syndicate members may get from alters’ direct participation in certain prior investments (the first network neighborhood), while structural equivalence captures the redundancy of information that alters may get from *their* alters and may pass on to the syndicate (the second network neighborhood). We will explore how redundancy in the second network neighborhood may moderate the value of redundancy in the first neighborhood.

Third, in relation to closed-diverse networks, closure may have two separate positive effects: the augmented effort of investors with shared third parties and the ability to triangulate interpretive cues. We will try to disentangle these two explanations empirically by introducing a control for tie strength, which should correlate more with the first advantage than with the second.^1^

Fourth, we portray open-specialized and closed-diverse networks as equally advantageous because both combine ease-of-interpretation and diversity advantages. But open-specialized networks offer within-sector diversity, whereas closed-diverse networks offer diversity from across sectoral boundaries. Starting from the notion that within-sector diversity may be more valuable for early-stage ventures in emerging industries, and between-sector diversity may be more valuable for ventures in established sectors, we explore how these two types of diversity may be advantageous for the success of different types of firms.

## Method

### Data

To test our hypotheses, we drew on CrunchBase (www.CrunchBase.com), a public database that provides an almost complete overview of recent venture capital funding in the U.S. information technology (IT) and Internet industry (Block and Sandner, 2009; Alexy et al., 2012). CrunchBase data are the source used by TechCrunch, a popular blog and major information source on startups, especially in the IT and Internet sector. This database provides data on new ventures, entrepreneurs, and investors in U.S. high-tech industries, including funding histories and board compositions for both small private firms and large publicly listed corporations. Data on investors include information on business angels, large venture capital funds such as Sequoia Capital, and corporate venture capital funds such as Siemens Venture Capital. In contrast to VentureXpert, CrunchBase includes recently founded ventures and those that have not yet received funding, allowing for fine-grained and reliable longitudinal data on syndication networks. We collected our CrunchBase data in May 2014 and had information on 10,266 ventures that received funding in a total of 37,146 funding rounds from 5,032 unique investors (primarily venture capital funds).

Because we were investigating the impact of syndication networks on additional funding, for our regressions we considered only firms that had received a first round of funding; we also excluded firms with only one investor (i.e., non-syndicated investments). We used all data to construct our network measures. We limited the time frame of our analysis to ventures that received their first funding between 2005 and 2011, so that we had sufficient time left to construct network variables based on prior investments and sufficient data from more recent years to observe ventures’ success events. Our final dataset included 2,371 syndicated first-round investments involving 1,646 unique investors.

In addition, to create some of our control variables, we constructed trademark portfolios based on U.S. Patent and Trademark Office data, and we obtained patent data from the PATSTAT database (version October 2013), provided by the Organisation for Economic Co-operation and Development (OECD) and the European Patent Office, which contains all patent applications and patents granted worldwide.

### Dependent Variable

Our interest was in analyzing the effect of investors’ network structure and actors’ knowledge similarity on venture success. We defined *venture success* as the venture’s ability to attract a second round of funding. Firms active in high-tech sectors typically require several rounds of funding at comparatively short intervals to support the development and diffusion of their products and services (Gompers, 1995). Thus receiving a second round of funding is a clear and positive signal either that the initial investors are happy with the venture’s progress and are willing to contribute additional resources or that the venture has been able to attract new, possibly larger and more experienced investors (e.g., Lerner, 1994; De Clercq et al., 2006). Although our sample included ventures that received first-round investment between 2005 and 2011, we observed second-round funding up to the end of 2013, which allowed us to observe funding events even for the most recent companies given an average time of 19 months between first- and second-round funding. CrunchBase data are updated frequently and have been shown to be accurate, so we are confident that we did not miss any second-round funding events that occurred before the end of 2013.

### Independent Variables

Our independent variables relate to the syndicate’s social capital at the time of first-round investment in the focal venture. Following the approach in Oh, Chung, and Labianca (2004), we operationalized syndicate social capital as the aggregate ego-level network of the syndicate group. Thus the syndicate’s social capital includes prior syndication relationships among syndicate members, additional prior syndication partners of individual members, and potential alter–alter ties between these partners. Ties are defined to exist between all investor pairs that co-invested in a venture in the five years up to and including the month prior to the focal investment. In the Online Appendix (http://asq.sagepub.com/supplemental), figure A1 depicts the interplay between different network structures and actors’ knowledge similarity in investment syndicates. Investors A, B, and C syndicate a first-round investment in venture X at time *t.* In all network configurations, A–B, A–C, and B–C have prior co-investments in the preceding five-year period. Also, A previously syndicated with D and E, B with E and F, and C with G. The prior syndication relationships between nodes D, E, F, and G differ between open and closed networks. Our network closure and actors’ knowledge similarity variables are calculated on the aggregate network structure of nodes A to G.

Starting with our structure-related variables, *network closure* is a local density measure computed as the number of ties among network actors over the maximum number of possible ties, $\frac{N*\left(N-1\right)}{2}$, where *N* is network size. The measure ranges from 0 (fully open network) to 1 (fully closed network) (see also Obstfeld, 2005; Fleming, Mingo, and Chen, 2007). In the lower two quadrants in figure A1, there are eight ties among 21 potential ties, yielding a closure value of 0.38. We ran alternative specifications of our models with measures for bridging ties, structural equivalence, and tie strength. *Bridging ties*—those that span a structural hole—are measured as the proportion of the syndicate’s links to prior partners that are bridging ties: ties to partners with no indirect ties through mutual contacts (Burt, 2010). Ties are also considered bridging if two syndicate members are tied to the same prior partner. *Structural equivalence* is defined as the extent to which alters of a node (in our case, the syndicate) overlap in their links to third parties, and it captures redundancy among alters’ information sources (Lorraine and White, 1971; Reagans and Zuckerman, 2008). For each alter, we compiled a list of third-party partners in the five years before the focal investment. We computed a Jaccard coefficient of overlapping partners for each pair of alters. Our measure of equivalence is expressed as the average pairwise coefficient across all pairs. Finally, *tie strength* is a dummy variable indicating that the investors in the network have syndicated with each partner at least twice on average.

*Actors’ knowledge similarity* is obtained on the basis of the past investment portfolios of all network actors, based again on their investments during the five years preceding the focal investment. The technological specialization of each investor in the network is described by a vector of length 32, where a cell describes which fraction of investments was in startups in a specific subsector of the IT industry. Actors’ knowledge similarity is calculated as the average cross-product of the vectors for each pair of network actors (Bonacich, 1972). In the two left-side quadrants in figure A1, all actors each invested 100 percent in blue, except for node B, which invested 50 percent in blue and 50 percent in green. It follows that the vector cross-products for the six dyads involving B take the value 0.5 while the remaining 15 cross-products take the value 1, yielding an average actors’ knowledge similarity score of [(6 × 0.5) + (15 × 1)] / 21 = 0.857. The actors’ knowledge similarity measure ranges from 0 (fully diverse network) to 1 (fully specialized network).

### Control Variables

Our control variables are organized on three levels. The venture-level set includes the log of the *amount raised* from the first round of funding, which allows us to control for the initial startup conditions such as the founder’s social capital and perceived quality of the idea. Shane and Stuart (2002: 160) argued that if the effect on the first round of funding is properly accounted for, these initial conditions will be of little importance for subsequent funding rounds. We also included cumulative counts for *patents* and *trademarks*, which are important quality signals (e.g., Baum and Silverman, 2004; Audretsch, Bönte, and Mahagaonkar, 2012; Block et al., 2014) and may have some liquidation value should the startup fail, both aspects that might increase investors’ propensity to invest (Tyebjee and Bruno, 1984; Shane and Stuart, 2002; De Clercq et al., 2006). For trademarks and patents, we relied on applications for intellectual property protection rather than granted rights because granted rights often involve a lengthy process and investors typically do not wait for their conclusion. We identified all the legal applicants associated with all ventures in our sample and accounted for misspelled names and complex organizational structures such as subsidiaries with different names or multiple legal entities. We set the value for patents and trademarks to 0 for ventures for which we found no data. Other venture-level controls included *year of first funding* to account for cyclical effects (six dummies) (Cumming, Fleming, and Schwienbacher, 2005), *IT subsector* in which the venture is specialized (31 dummies), and three *location* dummies for U.S. states with high concentrations of IT firms (California, New York, Massachusetts) to account for the geographic clustering of investments (see Sorenson and Stuart, 2001; Chen et al., 2010).

The second set of control variables is at the investor level. To assess the potential effect of overall quality or reputation of the first-round syndicate members (Gu and Lu, 2014), we included the *share of past portfolio companies that were acquired, or held an initial public offering (IPO)*. We also obtained the *venture capital reputation index* as calculated annually by Lee, Pollock, and Jin (2011), but because that variable was not available for all the investors in our data, we excluded it from our models. Its inclusion does not affect our findings. In line with extant research (Podolny, 2001; Shipilov and Li, 2008), we computed eigenvector centrality to measure *investor status*. As this variable is dependent on network size, we standardized it by total number of network actors (i.e., all syndicate members and their prior partners). We also included dummy variables for *angel investors* and *corporate venture capitalists.*

The third set of controls is at the level of the syndicate and its network. We included *syndicate size* because larger syndicates might have larger pools of resources, which might positively affect the venture’s chances of attracting a second round of funding (Lerner, 1994). We included a measure for *network size* without double-counting the contacts that multiple syndicate members have (i.e., both A and B are connected to E). In the examples in figure A1, this variable takes the value 4 for the syndicate’s relations to D, E, F, and G.

### Estimation Method

To estimate the effect of syndication network properties on venture success, we employed three estimation techniques. First, we used standard logit regression techniques with the venture–investor combination as the unit of analysis. Although this method does not account for potential censoring issues, the lagged structure of our dependent variable means these should be minimized. Also, logit models are generally well suited to estimating discrete-time events (Allison, 2010). Setting our unit of analysis at the venture–investor level allowed us to account accurately for the non-independence of observations that arose because ventures and investors occurred multiple times in our dataset, by estimating robust standard errors and clustering them at the venture and investor levels simultaneously (Cameron, Gelbach, and Miller, 2011; Kleinbaum, Stuart, and Tushman, 2013).^2^ We repeated all the estimations at the venture–syndicate level of analysis, which has the advantage that it gives equal sampling weight to each venture–syndicate observation regardless of syndicate size (rather than sampling *N* times each venture with syndicate size *N*). Our results are robust to shifting to the venture–syndicate unit of analysis. Likewise, adding sampling weights—the inverse of syndicate size—to the venture–investor-level analyses did not affect our estimates, suggesting that the sampling issue does not bias our findings. We prioritize the non-interdependence of observations at the investor level and report the venture–investor-level analyses in the paper.

Second, we employed piecewise exponential regressions with occurrence of and time to second-round funding as the dependent variable. We could not use a standard Cox model because the proportionality assumption was violated. The piecewise exponential method allows accurate estimation of timing effects and censoring and truncation issues, but because these models are at the venture–syndicate level, they do not account for the possibility of investors influencing multiple observations through involvement in multiple syndicates. Logit models remain our preferred specification, partly also because it is ambiguous whether timing until further funding always signals success. For example, entrepreneurs may purposefully delay further funding to resolve uncertainties in a bid to achieve a higher valuation or to retain more equity in the next round (Hallen and Eisenhardt, 2012).

Third, we ran Heckman probit selection models to account for the possibility that our results are driven by the ability of investors with high social capital to select more promising ventures. We adopted the approach in Hallen (2008: 717) and matched each syndicate that made a first-round investment in a particular venture to ten random alternative ventures that received first-round investment in the same year but from a different syndicate. After matching the venture–syndicate dyads, we expanded the database to the venture–investor level as in the main analysis. This makes for a better comparison and allows standard errors to be clustered at the venture, investor, and syndicate levels to account for the non-independence of observations. Our instrument is mean geographic distance between syndicate and venture. We believe that the proximity of a venture may make syndicate members more aware of some particular investment targets than others and thus affects selection. But we also believe that geographic distance has a negligible effect on the odds of successful further funding of a venture that already received first-round funding. In our sample, the difference in second funding success between firms at below-median levels of distance from their investors (*p* = .52) and those at above-median levels (*p* = .49) is marginal.

## Results

### The Interplay between Network Closure and Actors’ Knowledge Similarity

Table 1 provides summary statistics and correlations for all the variables included in the regressions. About half of the 2,371 ventures in the dataset that received first-round funding attracted a second round of funding, with a mean of 575 days between rounds. First-round syndicates have a mean size of 2.8 members and collectively have a mean beyond-syndicate network size of 74 investors. Table 2 shows that all four network configurations we investigated occur frequently in the dataset.

**Table 1.** Descriptive Statistics and Pairwise Correlations*

Variable	Mean	S.D.	Min	Max	1	2	3	4	5	6	7	8	9	10	11	12	13
*Venture*																	
1. Second funding received	.51		.00	1.00													
2. Raised amount first round (log)	1.22	.88	−1.39	2.30	.04												
3. Number of patents	.87	5.07	.00	112.00	−.02	.13											
4. Number of trademarks	2.07	6.80	.00	220.00	−.07	.11	.29										
*Investor*																	
5. Past portfolio companies acquired/IPO	.02	.07	.00	1.00	.03	−.01	−.05	−.02									
6. Status (eigenvector centrality)	.00	.00	.00	.02	.05	.15	.02	.00	.06								
7. Corporate venture capitalist	.05		.00	1.00	−.02	−.07	−.01	.00	.02	−.06							
8. Angel investor	.03		.00	1.00	.00	−.24	−.03	−.02	.01	−.03	.02						
*Syndicate*																	
9. Syndicate size	2.84	1.21	2.00	13.00	−.01	−.11	.08	.02	.00	−.22	.04	.07					
10. Network size	73.82	70.28	.00	381.00	.06	−.04	−.04	−.02	.26	−.03	.03	.08	.38				
11. Network closure^†^	.17	.11	.00	1.00	−.03	−.06	−.03	−.03	−.06	.07	.00	−.01	−.25	−.45			
12. Network specialization^†^	.11	.07	.00	1.00	.02	.19	.06	.01	−.08	.11	−.03	−.08	−.06	−.16	.48		
13. Bridging ties (proportion)^†^	.03	.08	.00	1.00	−.05	−.06	−.02	−.01	−.08	−.01	−.02	.02	−.13	−.24	.26	.10	
14. Structural equivalence (of alters)^†^	.02	.02	.00	.70	.03	−.09	−.02	−.04	.07	.10	.03	.01	−.15	−.10	.40	.00	.02

* Correlations above |0.03| are significant at 5% level. N = 2,371 for venture and syndicate variables; N = 1,646 for investor variables.

† Descriptive statistics show non-standardized variables, but variables were standardized before computation of correlations and inclusion in regression models.

**Table 2.** Co-occurrence of Low and High Levels of Network Closure and Actors’ Knowledge Similarity

Network closure	Actors’ knowledge similarity		
	< Mean (diverse networks)	≥ Mean (specialized networks)	Total
< Mean (open networks)	909	457	1,366
≥ Mean (closed networks)	517	488	1,005
Total	1,426	945	2,371

Table 3 presents the logit regression analysis. Model 1 includes only the venture-level covariates. Investor-level and syndicate-level control variables are introduced in models 2 and 3, respectively. Model 4 includes network closure and actors’ knowledge similarity, neither of which have significant independent effects on a venture’s success. Model 5 includes the interaction term between network closure and actors’ knowledge similarity, which is negative and significant, providing support for our hypotheses.

**Table 3.** Predicting Probability of Receiving Second Funding (Network Closure × Actors’ Knowledge Specialization)*

Variable	Model 1 Logit venture controls	Model 2 Logit investor controls	Model 3 Logit network controls	Model 4 Logit main effects	Model 5 Full model (preferred specification)	Model 6a Sample split: diverse networks	Model 6b Sample split: specialized networks	Model 7 Piecewise exponential full model	Model 8 part 1 Heckprobit selection equation	Model 8 part 2 Heckman probit success equation
*Venture*										
Mean distance to investors									−.254	
									(.020)^**•••**^	
Raised amount 1st round	.117	.103	.072	.073	.066	.151	.008	.011	−.006	.044
	(.067)^**•**^	(.057)^**•**^	(.062)	(.063)	(.064)	(.074)^**••**^	(.194)	(.035)	(.023)	(.041)
Number of patents	.002	.002	.004	.004	.003	.022	.004	−.008	.005	.002
	(.010)	(.011)	(.011)	(.011)	(.012)	(.019)	(.011)	(.010)	(.006)	(.007)
Number of trademarks	−.044	−.044	−.043	−.043	−.043	−.016	−.071	−.023	.003	−.025
	(.017)^**•••**^	(.016)^**•••**^	(.015)^**•••**^	(.016)^**•••**^	(.016)^**•••**^	(.008)^**••**^	(.014)^**•••**^	(.008)^**•••**^	(.003)	(.009)^**•••**^
*Investor*										
Share of past portfolio companies acquired/IPO		1.254	.969	.962	.956	.467	1.636	1.706	.099	.587
		(.565)^**••**^	(.499)^**•**^	(.498)^**•**^	(.494)^**•**^	(.557)	(.647)^**••**^	(.370)^**•••**^	(.064)	(.304)^**•**^
Status (eigenvector centrality)		19.896	9.496	8.833	3.959	14.930	−1.267	31.001	−6.072	2.975
		(6.250)^**•••**^	(4.159)^**••**^	(4.700)^**•**^	(3.598)	(10.182)	(5.873)	(10.327)^**•••**^	(1.235)^**•••**^	(2.457)
Angel investor		.178	.176	.175	.180	.106	.590	.196	−.017	.112
		(.351)	(.332)	(.330)	(.322)	(.306)	(.574)	(.237)	(.033)	(.204)
Corporate venture capitalist		−.232	−.217	−.219	−.224	−.039	−.549	−.152	−.058	−.135
		(.207)	(.210)	(.209)	(.210)	(.181)	(.294)^**•**^	(.159)	(.016)^**•••**^	(.132)
*Syndicate*										
Syndicate size			−.077	−.074	−.077	.014	−.215	−.033	.014	−.049
			(.049)	(.049)	(.050)	(.072)	(.056)^**•••**^	(.027)	(.006)^**••**^	(.030)
Network size			.002	.002	.002	.002	.001	.001	−.000	.002
			(.001)^**••**^	(.001)^**•••**^	(.001)^**••**^	(.001)^**•**^	(.002)	(.001)^**••**^	(.000)^**•••**^	(.001)^**•••**^
Network closure				.025	.030	.134	−.131	−.010	−.020	.019
				(.063)	(.064)	(.057)^**••**^	(.062)^**••**^	(.016)	(.007)^**•••**^	(.038)
Actors’ knowledge similarity				.003	.061			.044	.018	.035
				(.050)	(.058)			(.011)^**•••**^	(.007)^**••**^	(.032)
Network closure × Actors’ knowledge similarity					−.035			−.023	.003	−.022
					(.007)^**•••**^			(.003)^**•••**^	(.002)*	(.004)^**•••**^
Constant	.226	.202	.539	.520	.555	−.087	.077	−7.994	.446	.476
	(.241)	(.247)	(.225)^**••**^	(.220)^**••**^	(.230)^**••**^	(.557)	(1.023)	(.133)^**•••**^	(.165)^**•••**^	(.186)^**••**^
N	6,744	6,744	6,744	6,744	6,744	3,374	3,370	9,149	74,184	6,744
Unique investors	1,646	1,646	1,646	1,646	1,646	1,067	1,016	1,646	1,646	1,646
Unique ventures	2,371	2,371	2,371	2,371	2,371	1,111	1,260	2,371	2,371	2,371
Log pseudo-likelihood	–4,446	–4,438	–4,424	–4,423	–4,417	–2,183	–2,169	–4,565		–24,575
Wald χ^2^	400.66	411.78	444.91	445.84	460.03	283.38	269.61			162.08
LR-test		16.62^**•••**^	28.92^**•••**^	0.86	11.93^**•••**^					
Correlation error terms success-selection equation										–.088
										(.053)^**•**^

• *p* < .10; ^**••**^*p* < .05; ^**•••**^ *p* < .01.

* Standard errors (shown in parentheses) are clustered by investor, venture, and syndicate size in all models except model 7, in which standard errors are clustered on syndicate size only. Year dummies (2006–2011), location dummies (CA, MA, and NY), and dummies for the 32 IT subsectors are included and are jointly significant in all models. Time effects in model 7 are not shown. The unit of analysis is the venture–investor combination in all models, except model 7, in which it is the venture–syndicate combination. The sample split in models 6a and 6b is at sample median values. The sample includes firms that received first-round investment between $250,000 and $10 million between 2005 and 2011. Second funding is observed until the end of 2013. LR-test assesses improvement in model fit relative to model in previous column and is based on the log likelihood of the model with clustered standard errors on investor and venture levels only.

This result holds in both alternative specifications of the full model: taking account of timing and censoring effects (model 7) and controlling for selection (model 8). In the latter case, the marginally significant (at 10 percent) correlation between the error terms of the selection and venture success equations demonstrates that the selection process and the path to venture success are not completely independent. Controlling for the determinants of selection, however, the sign and significance of the coefficients in the venture success part of the model are consistent with the findings from model 5.

**Figure 3.**
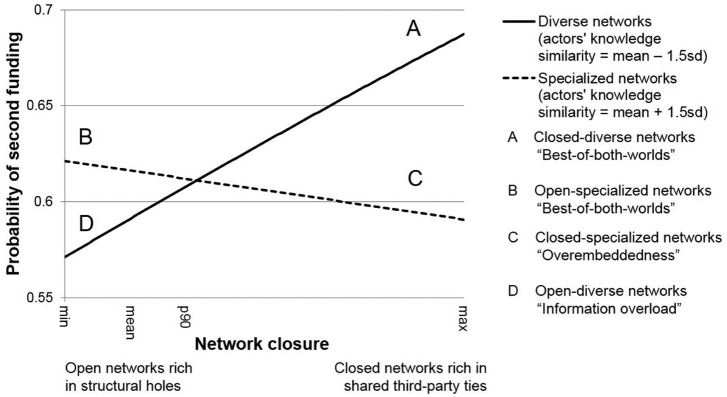
Interaction between network closure and actors’ knowledge similarity.* * The figure is based on the Heckprobit selection model, model 8 in table 3. Year is set to 2005, sector to mobile, and location to California. All remaining variables are set to sample mean. Note that using different values for control variables would only shift the regression lines upwards or downwards. Graphs based on models 5 (standard logit) and 7 (piecewise exponential) are highly consistent with the graph shown.

To gauge the nature of the interplay between network structure and actors’ knowledge similarity, and to illustrate the magnitude of the effects, figure 3 plots the interactions for a venture in the mobile communications sector in California, which received first-round funding in 2005. We explored the use of simulation techniques suggested by Zelner (2009) to graph interaction effects and confidence intervals, but this method does not support the multiple clustering of standard errors. Graphs obtained by clustering at only one level show that the difference in the predicted probability associated with a change in actors’ knowledge similarity from −1.5 to 1.5 (as depicted in figure 3) is statistically different from 0 (95 percent confidence) at either end of the network closure range but not in the middle of the range where the lines intersect.

We also conducted a sample split at median values of actors’ knowledge similarity (models 6a and 6b) and found consistent support for H1a and H1b. In line with H1a, figure 3 shows that ventures have a higher probability of success if their syndicates have closed-diverse networks (A) rather than closed-specialized (C) or open-diverse networks (D). The higher probability of the success of A relative to D is further supported in model 6a, which shows a positive association between network closure and venture success in diverse networks. In line with H1b, we found support also for our prediction that ventures benefit more from open-specialized syndication networks (B) than from closed-specialized networks (C) or open-diverse networks (D). The higher probability of venture success associated with B relative to C is supported by a negative association between closure and venture success in specialized networks in model 6b. These two findings taken together imply that, in line with H1a and H1b, there is a positive relationship between network closure and venture success if the actors are diverse and a negative relationship between network closure and venture success if the network actors are specialized.

To further test the robustness of our results, we conducted several additional analyses, not included here for reasons of space. First, to check for potential multicollinearity issues, we estimated a variant of model 5 that included only the investor social capital variables and the interaction term. The sign and significance level of the coefficients are largely unchanged, suggesting that multicollinearity is not a concern in our estimations. Second, we reran the logit models at the venture–syndicate rather than the venture–investor level of analysis, with investor-level covariates averaged across syndicate members. Although this setup does not allow us to control for investor-level interdependence, it enables a neater juxtaposition of the dependent variable at the venture level and the network explanatory variables at the syndicate level, and it leads to a more balanced sampling of ventures’ outcomes independent of syndicate size. The results are consistent with those reported in the paper. Finally, although five-year time windows are common in syndication network research (Sorenson and Stuart, 2001, 2008), we reran the models with shorter (one-year and three-year) and longer time spans (no tie decay) for our network variables. The results were consistent with those reported here.

### Structural Holes, Structural Equivalence, and Industry Emergence

Our results show that ventures have the highest chance of success if their syndicates have either closed-diverse or open-specialized networks. These findings support our theoretical argument that these configurations capture the best of both worlds, combining non-redundancy of information, which guarantees access to diverse information, and redundancy of information, which eases its interpretation. To probe whether these mechanisms are indeed driving our results, we conducted four further analyses.

**Figure 4.**
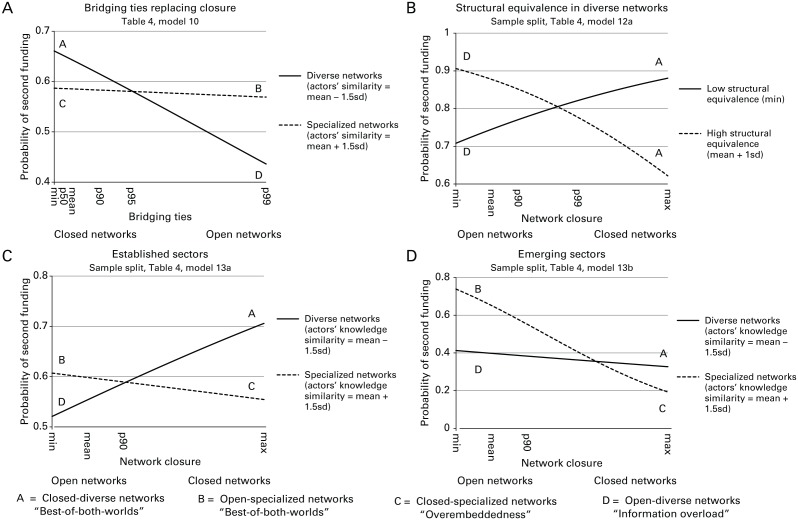
Bridging ties, structural equivalence, and industry emergence.* * Year is set to 2005, sector to mobile, and location to California. All remaining variables are set to sample mean. Note that using different values for control variables would only shift the regression lines upwards or downwards.

First, our argument is based in part on the theoretical assumption that open networks have more structural holes or bridging ties than closed ones. Although on average this is likely to be the case—the *count* of bridging ties and closure are correlated at −0.25—relatively open networks may have fewer structural holes if alter–alter ties are more widely distributed rather than being concentrated among selected groups of alters (see A and B in figure 2). In fact, the positive correlation between the *proportion* of bridging ties and closure suggests that the relation between closure and structural holes is not straightforward. In contexts such as ours, the distribution of bridging ties is skewed. Bridging ties are relatively rare because collaboration often takes places in larger groups, which create closure. To gauge the direct effect of bridging ties as a measure of non-redundancy, in models 9 and 10 of table 4, we replaced our closure variable with the proportion of bridging ties. Figure 4.A depicts the results. Consistent with our earlier results, we found that networks with low levels of bridging (i.e., closed networks) are associated with higher levels of venture success if these networks are diverse and that networks with high levels of bridging (i.e., open networks) are associated with higher levels of venture success if these networks are specialized. These results support our reasoning that structural holes are indeed an important mechanism driving our findings.

**Table 4.** Logit Models Predicting Probability of Receiving Second Funding (Structural Holes, Structural Equivalence, and Industry Emergence)*

Variable	Model 5 Full model for comparison	Model 9 Bridging ties main effect	Model 10 Bridging ties interaction	Model 11 Structural eqv.	Model 12a Structural eqv. diverse networks (compare 6a)	Model 12b Structural eqv. specialized networks (compare 6b)	Model 13a Sample split: established sectors	Model 13b Sample split: emerging sectors
*Venture*								
Raised amount 1st round	.066	.047	.054	.062	.114	.019	.100	−.041
	(.064)	(.073)	(.072)	(.067)	(.068)^**•**^	(.185)	(.058)^**•**^	(.097)
Number of patents	.003	.004	.004	.003	.021	.004	.003	
	(.012)	(.011)	(.011)	(.012)	(.019)	(.011)	(.011)	
Number of trademarks	−.043	−.046	−.047	−.043	−.016	−.070	−.044	
	(.016)^**•••**^	(.018)^**•••**^	(.018)^**•••**^	(.016)^**•••**^	(.008)^**••**^	(.015)^**•••**^	(.015)^**•••**^	
*Investor*								
Share of past portfolio companies acquired/IPO	.956	.867	.854	.956	.387	1.653	.708	1.322
	(.494)^**•**^	(.475)^**•**^	(.473)^**•**^	(.501)^**•**^	(.496)	(.671)^**••**^	(.384)^**•**^	(.972)
Status (eigenvector centrality)	3.959	−1.448	−.447	.147	−9.325	−6.169	−.291	20.852
	(3.598)	(3.984)	(4.185)	(2.413)	(15.112)	(7.078)	(5.492)	(14.996)
Angel investor	.180	.175	.190	.187	.132	.660	.050	.700
	(.322)	(.334)	(.322)	(.327)	(.295)	(.653)	(.400)	(.542)
Corporate venture capitalist	−.224	−.185	−.186	−.233	−.067	−.547	−.288	.115
	(.210)	(.188)	(.187)	(.203)	(.178)	(.286)^**•**^	(.244)	(.208)
*Syndicate*								
Syndicate size	−.077	−.093	−.092	−.075	.009	−.212	−.070	−.070
	(.050)	(.053)^**•**^	(.053)^**•**^	(.054)	(.073)	(.056)^**•••**^	(.041)^**•**^	(.102)
Network size	.002	.002	.002	.003	.003	.001	.003	−.001
	(.001)^**••**^	(.001)^**•**^	(.001)^**•**^	(.001)^**•••**^	(.001)^**••**^	(.002)	(.001)^**••**^	(.002)
Network closure	.030			−.017	−.054	−.140	.072	−.148
	(.064)			(.074)	(.129)	(.064)^**••**^	(.073)	(.045)^**•••**^
Actors’ knowledge similarity	.061	−.064	−.082	.063			−.000	.347
	(.058)	(.044)	(.039)^**••**^	(.054)			(.061)	(.089)^**•••**^
Network closure × Actors’ knowledge similarity	−.035			−.027			−.029	−.073
	(.007)^**•••**^			(.009)^**•••**^			(.011)^**•••**^	(.042)^**•**^
Bridging ties		−.092	−.106					
		(.038)^**••**^	(.061)^**•**^					
Bridging ties × Actors’ knowledge similarity			.060					
			(.027)^**••**^					
Structural equivalence				.100	.392	.150		
				(.040)^**••**^	(.159)^**••**^	(.131)		
Network closure × Structural equivalence					−.131	−.026		
					(.025)^**•••**^	(.036)		
Constant	.555	.846	.834	.631	.520	1.452	.561	−.877
	(.230)^**••**^	(.298)^**•••**^	(.302)^**•••**^	(.206)^**•••**^	(.502)	(.354)^**•••**^	(.194)^**•••**^	(1.030)
N	6,744	6,410^**†**^	6,410^**†**^	6,744	3,374	3,370	5,132	1,612
Unique investors	1,646	1,439	1,439	1,646	1,067	1,016	1,365	744
Unique ventures	2,371	2,229	2,229	2,371	1,111	1,260	1,805	566
Log pseudo-likelihood	–4,417	–4,209	–4,205	–4,412	–2,159	–2,169	–3,359	–1,026
Wald χ^2^	460.03	404.50	402.23	466.71	326.42	264.67	346.92	174.87

• *p* < .10; ^**••**^*p* < .05; ^**•••**^*p* < .01.

* Standard errors (shown in parentheses) are clustered by investor, venture, and syndicate size in all models. Year dummies (2006–2011), location dummies (CA, MA, and NY), and dummies for the 32 IT subsectors are included and are jointly significant in all models. The unit of analysis is the venture–investor combination. The sample includes firms that received first-round investment between $250,000 and $10 million between 2005 and 2011. Second funding is observed until the end of 2013. Ventures in emerging sectors in our sample have no patents or trademarks.

† Some observations were dropped, as the proportion of bridging ties is undefined when all network relations are contained within the syndicate.

Second, another challenge to closure as a measure of redundancy relates to structural equivalence. Our argument is based on the notion that closure among a syndicate’s alters is indicative of information redundancy in the network. Although this will hold on average, there may be cases in which alters, despite being unconnected, have mostly the same information because they are tied to the same third parties, that is, they are structurally equivalent. Model 11 tests whether our results hold if we add structural equivalence to our original specification of model 5. The model shows that controlling for redundancy based on structural equivalence does not affect our main result; the interaction effect between network closure and actors’ knowledge similarity is substantively unchanged. Overall, structural equivalence among alters has a positive and significant effect on venture success, suggesting that some overlap in the information that alters pass to the syndicate may be beneficial for its interpretation. This might be because overlap in the second network neighborhood increases the chances that certain bits of information reach the syndicate through different routes (and potentially in different versions), which allows the syndicate to interpret the information through triangulation.

But the value of the structural equivalence of alters—that is, redundancy in the second network neighborhood—may just as well be contingent on the redundancy emanating from closure and actors’ knowledge similarity in the first network neighborhood. Models 12a and 12b are sample split analyses that mimic the approach in models 6a and 6b. There, we see how the value of closure in diverse or specialized networks may depend on the level of structural equivalence. One would expect that the value of closed-diverse networks to new ventures might be reduced if structural equivalence is high. If the syndicate’s alters get their information largely from the same sources, information diversity might be less than one would conclude from considering only closure among the alters. Conversely, in open-diverse networks, problems of information overload due to excessive non-redundancy might be mitigated if structural equivalence among alters is high. Despite the alters not being directly connected, information diversity is limited because they rely on the same third-party sources. Model 12a and figure 4.B support these intuitions.

Similar reasoning can be applied to specialized networks. The value of open-specialized networks could be compromised if the syndicate’s unconnected alters use the same third-party sources. The non-redundancy emanating from a sparse first neighborhood structure is limited if alters are structurally equivalent. Also, we might expect that problems of overembeddedness are exacerbated if redundancy is also high in the second neighborhood of the network. But model 12b does not support this reasoning. Although the main effect of closure in a specialized network remains negative—keeping our evidence for the value of open-specialized networks intact—the level of structural equivalence appears neither to reduce the advantages of open-specialized networks nor to reinforce the disadvantages of closed-specialized ones.

Third, we have argued that the advantage of closure in closed-diverse networks and the disadvantage of a lack of closure in open-diverse networks are based on two mechanisms: the known mechanism that cohesion of shared third parties around a tie pushes actors to put more effort into the exchange (Reagans and McEvily, 2003) and our newly proposed mechanism that closure among diverse actors enables the triangulation of diverse interpretive cues of the same information. Controlling for tie strength in model 6a does not reduce the magnitude or significance of the positive effect of closure in diverse networks. Assuming the tie strength variable to be a more direct measure of augmented effort, we consider this result indirect evidence that the logic of triangulation we proposed indeed plays an important part in the advantages derived from closed-diverse networks and disadvantages of open-diverse networks.

Finally, we have so far portrayed the two best-of-both-worlds network configurations—closed-diverse and open-specialized—as equally beneficial in terms of information advantages. To shed light on how the mechanisms driving advantage in these configurations differ, we explore whether the venture’s level of technological uncertainty, a crucial external contingency (e.g., Khandwalla, 1977), influences the relative impact of these two network structures. We exploited a change in the classification in CrunchBase data to distinguish between ventures in established and emerging sectors. Up to the end of 2013, CrunchBase employed 11 classes to categorize IT and Internet industry subclasses. These related mostly to the technical underpinnings of IT and included fields such as hardware, network hosting, search, and security, as well as relatively established areas of application such as e-commerce and video games. In 2014, CrunchBase introduced a more inclusive classification scheme including 32 sectors, and it retrospectively recoded all the ventures in its database. The 21 additional sectors refer mostly to new IT application areas, notably mobile apps. We labeled the 11 original categories *established sectors* and the 21 new ones *emerging sectors*. The proportion of first-round investments in emerging sectors in our sample increased from around 15 percent in 2005, our earliest observation year, to more than 30 percent in 2011, the last year observed.

In table 4, models 13a and 13b present the logit analysis for the sample split into ventures in established and emerging sectors. The interaction between network closure and actors’ knowledge similarity is negative and significant in both models. Figures 4.C and 4.D, however, show remarkable differences in the values for open-specialized and closed-diverse networks for ventures in emerging and established sectors, respectively. New ventures in established sectors are most likely to be successful if their syndicates have closed-diverse networks, while ventures in emerging sectors benefit most from syndicates with open-specialized networks. To be clear, for both types of ventures, the nature of the interplay remains unchanged: as in the full sample, the relationship between closure and success is negative in specialized networks and positive in diverse networks. But there is a shift in the point at which the lines describing the relationship between closure and venture success for specialized and diverse networks intersect.

The differences between the value of closed-diverse and open-specialized networks may be due to variety in the former configuration being based on diversity in insights from different domains and the latter being limited to variation within domains. Between-domain variety in closed-diverse networks may be particularly valuable in settings in which lock-in to established and taken-for-granted views is a potential risk (see Bruner, Goodnow, and Austin, 1956; Uzzi, 1997). At the same time, interpretation of between-domain variety would benefit from a collaborative approach with the involvement of shared third parties, which are numerous in closed-diverse networks. In contrast, within-domain variety accessed via open-specialized networks may be better suited to settings in which views have not yet become established (see Brown and Duguid, 1998). When there is little consensus about what might be good for firms within a particular field, a variety of views from experts with different experience within a particular field may be more valuable than different views from across field boundaries. Given that variety is mostly within-domain, independent interpretation without the involvement of shared third parties may be sufficient. This might explain our finding that open-specialized networks are particularly beneficial for ventures in emerging sectors.

## Discussion

We have argued that the information advantages of social capital are embodied in the combination of non-redundant information, which provides access to diverse insights, and redundant information, which eases their interpretation. These advantages can be derived from either closed networks among dissimilar actors or open networks among similar actors. We found that new ventures benefit most from the social capital of their investors and thus are more likely to be successful at attracting additional funding if their initial investors’ networks are closed-diverse or open-specialized. These two configurations are associated with higher levels of venture success than open-diverse networks, in which investors have limited means to interpret diverse information, and closed-specialized networks, in which the diversity of information is too limited.

### Redundancy, Non-redundancy, and Social Capital

In the literature on network structure, both redundancy and non-redundancy are considered pivotal to the information advantages derived from social capital, resulting in a longstanding debate over whether open structures with non-redundant information or closed structures with redundant information provide more valuable information (Ahuja, 2000; Burt, 2004). Our findings imply that to benefit from networks, actors need both redundancy and non-redundancy of information. Looking at only the dichotomy between open and closed structures cannot explain how these two properties are combined. By bringing actors’ knowledge similarity into the open-versus-closed-networks debate, we argue that redundancy can stem from the similarity of actors’ knowledge in open networks with non-redundant information, and non-redundancy can come from the dissimilarity of actors in closed networks with high levels of structural redundancy.

Informational advantages associated with social capital may be maximized when redundancy and non-redundancy of information coexist, because the former aids interpretation and the latter safeguards diversity. Actors may benefit from non-redundant information in open networks if they have some knowledge similarity with information providers that yields shared interpretive schema and creates redundancy between the received information and actors’ prior knowledge. Actors may benefit from non-redundant information in diverse networks if they can rely on joint third parties whose interpretation of the information from their perspective creates redundancy and enables triangulation. Conversely, combined non-redundancy from open networks and from actor dissimilarity can lead to problems of information overload in open-diverse networks, while combined redundancy from closed networks and from high similarity of actors’ knowledge leads to problems of overembeddedness (Uzzi, 1997).

Our findings support the growing consensus that network structure is insufficient to explain the value of social capital (Kwon and Adler, 2014). Social capital research has a long tradition of structuralist studies (Kilduff and Brass, 2010) that attribute much of the variance in performance outcomes to differences in network position and network structure but that may not sufficiently take into account explanations based on actors’ heterogeneity (Reagans and McEvily, 2003; Rodan and Galunic, 2004), network content (Chua, Ingram, and Morris, 2008; Sosa, 2011), or diversity in the information environment (Aral and Van Alstyne, 2011). We show that the value actors obtain from the network is a function of the actors’ structural positions, the similarity of their knowledge, and the interplay between these two factors, which suggests that the effects of network structure (or content, for that matter) cannot be studied in isolation. Actors’ knowledge similarity is an important contingency in the value of network structure and sheds light on the boundary conditions when structural holes and closure advantages apply.

### Interpreting Diverse Information in Networks

Network actors’ ability to interpret information is also an integral component of social capital. Rather than assuming that actors in open or diverse networks are able to interpret all the information they access, we have highlighted two network-level mechanisms that facilitate the effective interpretation of non-redundant or diverse information: overlap in actors’ knowledge profiles and triangulation via shared third parties.

The relative similarity in knowledge profiles among actors in open-specialized networks creates potential overlap between the information they receive and the information they may already have. This information redundancy makes it more straightforward to overcome potential interpretive barriers and surmount the limitations imposed by the lower transmission capacity typical of sparse network structures (Shannon and Weaver, 1948; Simon and Feigenbaum, 1964). Our argument offers new insights relative to extant research on structural holes in which the actors bridging such holes are often assumed to be able to combine and integrate knowledge and use it to their own advantage, regardless of their knowledge similarity to information providers. Although brokers may to some extent benefit from diverse information merely through the perspective-broadening effect it has on the “engaged mind” (Burt, 2010), this strand of work treats the interpretive ability of network actors largely as a nodal property that is exogenous to the network, and thus it usually falls short of theorizing the process explicitly (Burt, 2004).

Our results imply that structural holes may be expected to have negative effects in heterogeneous information environments and positive effects in settings in which the information environment is relatively homogeneous and the interpretation of relatively diverse information is less problematic. This is in line with earlier work (Burt, 2005) showing that bankers can improve their performance by spanning structural holes in a professional context in which—in our words—diversity among actors is low. We would encourage further research in different contexts to understand whether and how the value of structural holes is contingent on the level of actors’ knowledge similarity and the diversity of the information environment more generally.

Another mechanism facilitating the interpretation of diverse information is triangulation via shared third parties. When the diversity of information stems from the dissimilarity of the actors in a network rather than from structural holes, shared third-party connections act as an important mechanism that helps actors interpret information in the network. These arguments extend earlier work on actors’ knowledge similarity and diversity in closed networks. Reagans and McEvily (2003) hinted at the possibility that a combination of high network closure and diverse actors may be the optimal network structure, although they did not directly examine the interplay between these two factors and did not point to the value of the combination of similar actors in networks with low levels of closure. Similar to Tortoriello and Krackhardt’s (2010) findings in the context of ties across intra-organizational boundaries, we find that the advantages of diversity from across domain boundaries are best realized in closed networks rich in shared connections.

Shared third parties not only encourage two connected actors to deepen the level of their exchange (Reagans and McEvily, 2003) but also may contribute directly to corroborating the information by providing a platform for collective interpretation (Gavetti and Warglien, 2015). If a focal actor receives the information directly from the original source and also receives the interpreted and adapted version from a shared dissimilar alter, that actor can triangulate the different versions of the same story and make inferences about his or her own interpretation (Weick and Roberts, 1993). Tightly knit groups of interconnected, dissimilar actors can function as a platform for distributed cognition allowing for the meaningful interpretation and application of even highly novel and unfamiliar information (Michel, 2007). This advantage from closure is specific to closed-diverse networks, because closed-specialized networks with homogeneous actors are unlikely to show major differences in the interpretation of the same information. In fact, the negative effect of closed-specialized networks on ventures’ success may be driven in part by the risks of groupthink, which emerge when the views of tightly interconnected groups achieve convergence without these views being challenged by relative outsiders (Janis, 1972). These findings relate directly to Uzzi’s (1997) discussion of overembeddedness as a disadvantage because inflowing novel perspectives are limited by an emphasis on strong, embedded ties in dense network structures. We add that this lack of inflow of diverse information will be particularly salient if high levels of actors’ knowledge similarity and high levels of network closure coincide.

### Sources of Diverse Information

Finally, we have portrayed various sources of diverse information. In our framework, diverse information is derived either from embeddedness in open networks rich in structural holes or from embeddedness in networks of heterogeneous actors. We build on a growing body of research on actors’ heterogeneity (Reagans and McEvily, 2003; Rodan and Galunic, 2004; Tortoriello, McEvily, and Krackhardt, 2015) and network content (Chua, Ingram, and Morris, 2008; Sosa, 2011) as additional sources of information diversity, and we challenge the assumption that actors’ knowledge diversity typically coincides with low levels of network closure (Reagans, Zuckerman, and McEvily, 2004) while actors’ knowledge similarity coincides with high levels of network closure (Uzzi, 1997). We found these two sources of diversity to be substitutes rather than complements. Problems of information overload occur when actors are faced with diverse information due to both actors’ heterogeneity and network structure. This finding contrasts with Rodan and Galunic’s (2004) study of managers’ networks, which suggests that actors’ heterogeneity and structural holes are mutually reinforcing sources of diversity.

Proposing network structure and actors’ knowledge similarity as distinct sources of information diversity raises questions about the extent to which structural holes and actors’ diversity capture the same type of information diversity. Despite finding network sparseness and actors’ heterogeneity to be mutually reinforcing, Rodan and Galunic (2004) also found that the main effect of structural holes on innovation disappears if a direct measure of diversity is included in the equation. This suggests that, in the context of their study, network sparseness functioned as a proxy for diversity. In our setting, however, the frequent occurrence of closed-diverse networks and open-specialized networks suggests that neither network openness nor closure is necessarily indicative of the level of actors’ knowledge similarity. Also, post-hoc analysis contrasting established and emerging sectors showed that the two sources of diversity are not perfectly equivalent. Closed-diverse networks are most strongly associated with venture success in established IT subsectors because they can offer cross-domain diversity that can help challenge established assumptions and taken-for-granted views. Open-specialized networks have within-domain diversity that appears particularly beneficial for ventures in emerging subsectors, in which assumptions and views are still emerging. These findings complement those of Bellavitis and colleagues (2014) who showed that extra-industry networks positively affect ventures’ performance, while intra-industry networks have a negative effect unless these networks are complemented by strong extra-industry ties.

We also shed light on the different roles of information diversity at different levels in the network. Although our story revolves mainly around the value of redundant and non-redundant information from the first neighborhood of the syndication network (syndicate members’ prior partners), we have also demonstrated how the value of such (non-)redundancy can be enhanced or reduced by (non-)redundancy in the second neighborhood (the third parties that inform the syndicate’s prior partners). We found that the advantages of closed-diverse networks are undermined if diversity is reduced by syndicate alters having highly overlapping third-party sources, while the problem of excess diversity in open-diverse networks is mitigated when equivalence is high. Accordingly, we suggest that the benefits of balance between structure and actors’ knowledge diversity documented for the first neighborhood network may also be attainable beyond that level. Future research could further investigate these tradeoffs.

### Suggestions for Future Research

Our study has several shortcomings that suggest directions for future research. First, our findings relate to the indirect effect of network structure and actors’ knowledge similarity on desired performance outcomes. Although the mechanisms we describe have strong validity based on prior work on the effects of investors’ social capital on venture performance (Hochberg, Ljungqvist, and Lu, 2007; Hallen, 2008), more work is needed to achieve a more fine-grained understanding of the type of advice syndicates bring to their portfolio companies and how it contributes to their success. For example, we provide indirect evidence that investor syndicates in closed-diverse networks benefit from the corroboration of diverse interpretive cues through triangulation, which affects the advice they give to ventures. Qualitative and experimental research could provide more evidence of this mechanism as a crucial driver of information advantage in closed-diverse networks. Also, although we tested for the alternative explanation that ventures with investor syndicates with the best-of-both-worlds networks are more successful because these networks allow the syndicates to select more promising ventures in the first place, we acknowledge that our approach may not have ruled out endogeneity entirely.

Second, various contextual factors are not accounted for in this study but have been highlighted in prior research on social capital as important contingencies related to the value of structural holes and network closure. There is a near consensus in the literature that structural holes are conducive to idea generation and knowledge creation and that network closure is beneficial for the implementation of ideas and innovation (Kilduff and Brass, 2010). Research has also suggested that closure and brokerage effects differ in relation to the time required for their manifestation (Soda, Usai, and Zaheer, 2004; Baum, McEvily, and Rowley, 2012). We do not claim to know the extent to which these explanations are compatible with our actors’ knowledge similarity approach.

Third, actors’ knowledge similarity is not exogenous to the network structure. Both network structure and actors’ knowledge similarity are based on the set of investments in the five years prior to the focal investment. Thus every syndicated co-investment creates a tie while also rendering the investors slightly more similar in their investment focus. Although this situation mimics reality—collaboration makes actors more similar over time (Cowan and Jonard, 2009)—the use of exogenously determined actors’ attributes might help to disentangle redundancy from network structural effects and redundancy related to actors’ attributes. Future research could use actors’ knowledge similarity measures based on, for example, text analysis of documents that characterize the actors’ knowledge profiles, which are separate from network data sources. It could also help to unravel the complex theoretical interdependencies between network structures and actors’ knowledge attributes that drive the formation of closed-diverse, closed-specialized, open-diverse, and open-specialized networks.

Finally, we confined the testing of our hypotheses to the specifics of syndication networks and their effect on ventures. Our study not only highlighted the value of closed-diverse and open-specialized networks relative to open-diverse and closed-specialized ones, it also pinpointed the difference in value between the two best-of-both-worlds network configurations for ventures in emerging versus established sectors. Although both closed-diverse and open-specialized networks combine diversity and interpretation advantages, the complex challenge of interpreting diverse information from across sectoral boundaries seems best handled in closed networks, which may not only be stronger (Hansen, 1999) but also offer built-in mechanisms for collective interpretation of unfamiliar or complex information. By contrast, the value of structural holes seems confined to more homogenous information environments contained within knowledge domains. We hope that our study offers a valuable and comprehensive theoretical framework with which future research can continue to explore, in a broader range of contexts, how the diversity (dis)advantages of open network structures and of actors’ knowledge heterogeneity and the interpretation advantages of network closure and actors’ knowledge homogeneity interplay. Although we would predict that the value of closed-diverse and open-specialized networks and the dangers of open-diverse and closed-specialized networks will apply to other settings, such as firms’ innovation alliances, it may be that some informational advantages as portrayed in this study may be particularly pertinent in the context of early-stage investments. In that context, social capital advantages can be expected to be strongly manifested because investors’ advice at that stage should have a formative impact on the venture itself, including its business model and organizational design, compared with later-stage investments or alliances between established firms, in which the focus may be on growing an existing idea to reach a more-refined technology development stage, new customer segments, or new geographic areas.

## Supplementary Material

Supplementary material

## Footnotes

1 We are indebted to one of our reviewers for this insightful suggestion.

2 Given that syndicate size might have a major influence on the values of our network variables, we clustered standard errors also by syndicate size. Specifically, small syndicates are more likely to have either high or low values of closure or actors’ knowledge similarity than intermediate values. Thus the standard errors of network effects should be non-independent of syndicate size.
