# Supplementary material for: The Best of Both Worlds: The Benefits of Open-specialized and Closed-diverse Syndication Networks for New Ventures’ Success
Source: Adm Sci Q. 2016 Mar 2;61(3):393–432. doi: 10.1177/0001839216637849 (PMC4959036; doi:10.1177/0001839216637849)
Supplement: Supplementary material [file DS_10.1177_0001839216637849.pdf]

ONLINE APPENDIX

Figure A1. The interplay between network structure and actors' knowledge similarity.

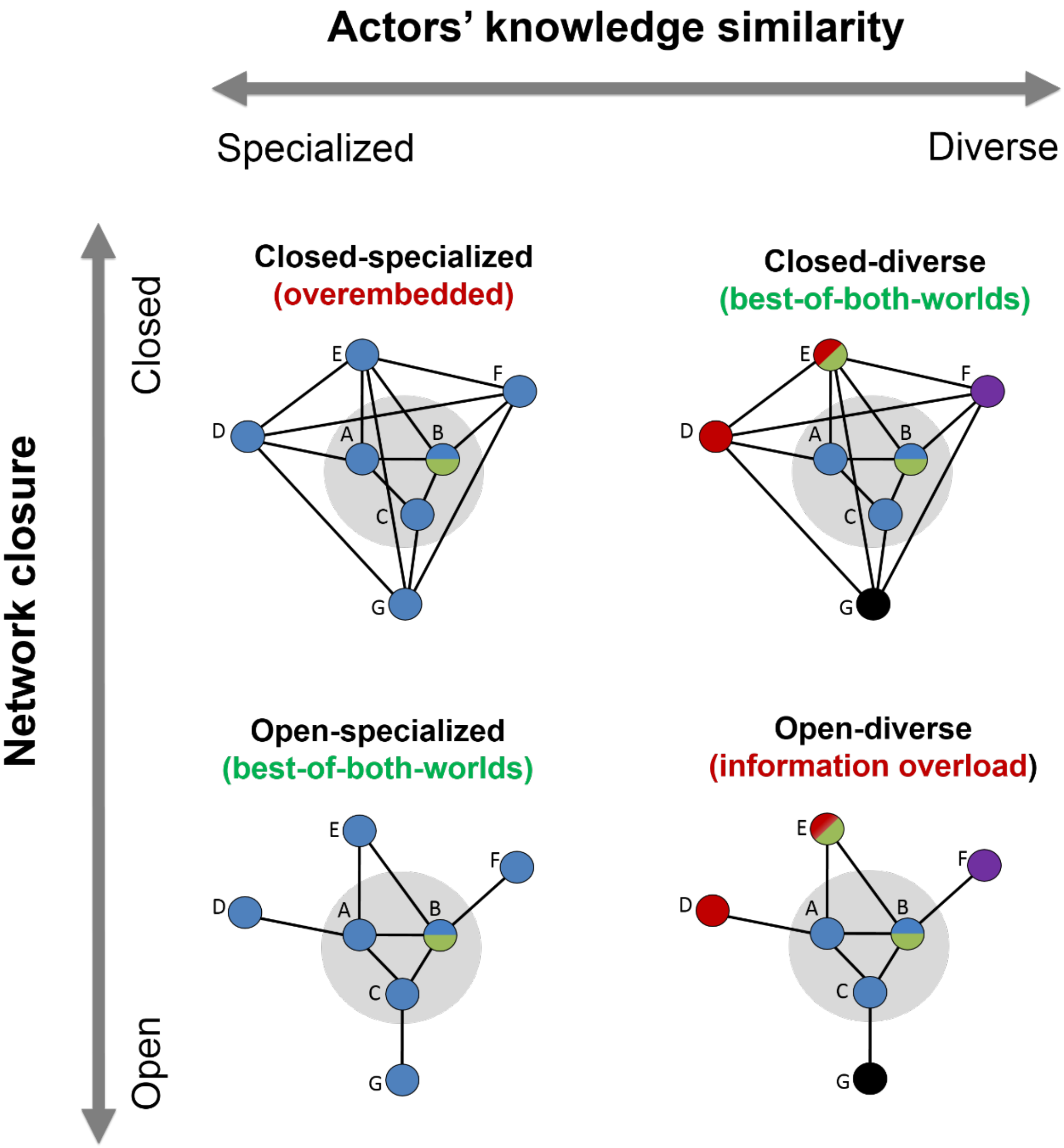

*** Node colors are indicative of the sectoral focus of actors' past investments. Nodes with multiple colors have prior investments in multiple sectors.**
